# Etiologic-sociodemographic assessment and comparison of dialysis modalities in pediatric Syrian migrants with chronic kidney disease

**DOI:** 10.1590/2175-8239-JBN-2020-0260

**Published:** 2021-08-16

**Authors:** Mehtap Çelakıl, Yasemin Çoban

**Affiliations:** 1 Hatay State Hospital, Pediatric Nephrology, Hatay, Turkey.; 2 Hatay State Hospital, Pediatric Intensive Care, Hatay, Turkey.

**Keywords:** Renal Insufficiency, Chronic, Dialysis, Child, Transients and Migrants, Syria, Insuficiência Renal Crônica, Diálise, Criança, Migrantes, Síria

## Abstract

**Background:**

Chronic kidney disease (CKD) and end-stage renal disease (ESRD) are among the important causes of mortality and morbidity in childhood. Early diagnosis and treatment of the underlying primary disease may prevent most of CKD patients from progressing to ESRD. There is no study examining chronic kidney diseases and dialysis modalities in Syrian immigrant children. We aimed to retrospectively research the etiologic, sociodemographic, and clinical factors in CKD among Syrian refugee children, and at the same time, to compare the clinical characteristics of patients with ESRD on peritoneal dialysis and hemodialysis.

**Methods:**

Our study included a total of 79 pediatric Syrian patients aged from 2-16 years monitored at Hatay State Hospital pediatric nephrology clinic with diagnosis of various stages of CKD and with ESRD. Physical-demographic features and clinical-laboratory information were retrospectively screened.

**Results:**

The most common cause of CKD was congenital anomalies of the kidneys and urinary tracts (CAKUT) (37.9%). Other causes were urolitiasis (15.1%), nephrotic syndrome (10.1%), spina bifida (8.8%), hemolytic uremic syndrome (7.5%), and glomerulonephritis (7.5%). Twenty-five patients used hemodialysis due to bad living conditions. Only 2 of the patients with peritoneal dialysis were using automatic peritoneal dialysis (APD), with 5 using continuous ambulatory peritoneal dialysis (CAPD). Long-term complications like left ventricle hypertrophy and retinopathy were significantly higher among hemodialysis patients. There was no difference identified between the groups in terms of hypertension and sex.

**Conclusion:**

Progression to ESRD due to preventable reasons is very frequent among CKD patients. For more effective use of peritoneal dialysis in pediatric patients, the responsibility of states must be improved.

## Resumo

**Histórico:**

Doença renal crônica (DRC) e doença renal em estágio terminal (DRET) estão entre as causas importantes de mortalidade e morbidade na infância. Diagnóstico precoce e tratamento da doença primária subjacente podem evitar que a maioria dos pacientes com DRC progrida para DRET. Não há estudos examinando doenças renais crônicas e modalidades de diálise em crianças imigrantes sírias. Visamos pesquisar retrospectivamente fatores etiológicos, sociodemográficos e clínicos na DRC entre crianças refugiadas sírias e, ao mesmo tempo, comparar características clínicas de pacientes com DRET em diálise peritoneal e hemodiálise.

**Métodos:**

Nosso estudo incluiu 79 pacientes pediátricos sírios com idades entre 2-16 anos monitorados na clínica de nefrologia pediátrica, Hospital Estadual de Hatay, com diagnóstico de vários estágios de DRC e com DRET. Características físico-demográficas e informações clínico-laboratoriais foram examinadas retrospectivamente.

**Resultados:**

A causa mais comum de DRC foram anomalias congênitas dos rins e trato urinário (CAKUT) (37,9%). Outras causas foram urolitíase (15,1%), síndrome nefrótica (10,1%), espinha bífida (8,8%), síndrome hemolítico-urêmica (7,5%), e glomerulonefrite (7,5%). 25 pacientes fizeram hemodiálise devido às más condições de vida. Apenas 2 dos pacientes em diálise peritoneal estavam usando diálise peritoneal automatizada (DPA), com 5 em diálise peritoneal ambulatorial contínua (DPAC). Complicações em longo prazo, como hipertrofia do ventrículo esquerdo e retinopatia, foram significativamente maiores entre pacientes em hemodiálise. Não identificou-se diferença entre grupos em termos de hipertensão e sexo.

**Conclusão:**

Progressão para DRET devido a razões evitáveis é muito frequente entre pacientes com DRC. Para utilização mais eficaz de diálise peritoneal em pacientes pediátricos, a responsabilidade dos estados deve ser aprimorada.

## Introduction

Chronic kidney disease (CKD) is among the problems with high mortality in childhood in the present days. According to the North American Pediatric Renal Trials and Collaborative Studies (NAPRTCS) and European studies, the most common causes of pediatric CKD are congenital anomalies of the kidney and urinary tract (CAKUT) (48-58%) and hereditary nephropathy (10-19%).^1^^,^ 2 Similarly, in Asian countries, after CAKUT (47-62%) and hereditary disease, obstructive uropathy is among the most common causes (17-30%).^3^^,^ 4 CKD is defined as abnormalities of kidney structure or function, present for >3 months, with implications for health. CKD is classified based on cause, glomerular filtration rate, and albuminuria category.^5^^,^ 6 Due to frequent causes of disease being treatable and preventable, early diagnosis and appropriate treatment is very important in terms of patient prognosis and reducing the financial load on the state. This is possible with easy access to health organizations and institutions. In Turkey, the number of pediatric patients who are Syrian refugees is increasing every year since the beginning of the civil war in Syria in 2011. Due to frequent intermarriage in the population, hereditary and congenital kidney diseases are observed more.^7^^,^ 8

Basic needs such as shelter, food, and health care, and also education, social activities, and employment opportunities are provided by the Turkish government. There are official sworn translators in all hospitals affiliated with the state for language problems. Thus, language-related problems in doctor-patient communication are eliminated. All healthcare needs, including diagnosis and treatment, are met free of charge in all state and university hospitals in our country for all Syrian immigrant patients. In spite of free access to health care in our country, late attendance at health centers due to a variety of socioeconomic and psychological factors causes delays in the diagnosis and treatment of these patients.

In our study, we aimed to retrospectively research the etiologic, sociodemographic, and clinical factors in CKD among Syrian refugee children, and at the same time, to compare the clinical characteristics of patients with end-stage renal disease (ESRD) on peritoneal dialysis and hemodialysis.

## Materials and Methods

Our study included a total of 98 pediatric Syrian patients aged from 2-16 years monitored from September 2019 to September 2020 at Hatay State Hospital pediatric nephrology clinic with diagnosis of various stages of CKD (stage 1-4) and with ESRD continuing hemodialysis or peritoneal dialysis treatment. Physical features like patient age, sex, height, and weight; demographic features like consanguinity, family kidney disease history, number of siblings and socioeconomic level; and clinical-laboratory information (urea, creatinine, electrolyte values, blood biochemistry, urine tests, urinary ultrasonography, voiding cystoureterography (VCUG), DMSA results, renal biopsy results) were retrospectively screened. Nineteen patients without follow-up or with missing data in files were removed from the study. The study included 47 patients with stage 1-4 CKD, and 25 patients with hemodialysis and 7 patients with peritoneal dialysis for ESRD (total of 79 cases). CKD was defined based on the Kidney Disease Outcomes Qualitative Initiative and the International Study of Kidney Disease in Children guideline. Primary diseases underlying CKD and biopsy results, in patients with renal biopsy performed, were recorded.

### Statistical Analysis

Statistical analysis was performed with IBM SPSS 20.0 (IBM Corp., Armonk, NY, USA) program. The Kolmogorov-Smirnov test was used to assess the fit to normal distribution. As data did not follow a normal distribution, numerical variables are given as median (25^th^-75^th^ percentiles). Categorical variables are expressed as frequency (percentage). Differences between groups were determined with the Mann Whitney U test as the normal distribution assumption was not met. Correlations between categorical variables were assessed with the chi-square analysis. To test two-way hypotheses, p<0.05 was accepted as sufficient for statistical significance.

## Results

Of the 79 patients included in the study, 45 were male (56.9%) and 34 were female(43.1%). Thirty-two patients had end-stage renal failure with 25 in hemodialysis and 7 in peritoneal dialysis. Median age at diagnosis was 8.4 years (minimum 2, maximum 16 years). The male/female ratio was 48/31 (1.54). There was consanguinity history in 51 patients (64.5%). There were 36 patients (45.5%) with kidney disease history in at least one sibling. The number of patients receiving diagnosis in the antenatal period was just 2 (2.5%). The distribution of diseases causing CKD is given in Table 1. Accordingly, the most common cause was CAKUT (37.9%). The most common disease within CAKUT was ureteropelvic (UP) stenosis (Table 2). The male patient rate was higher in the CAKUT group (M: 21, F: 9), with the number of girls with urolithiasis (F: 8, M: 4) significantly higher (*P*<0.05). There was no significant difference in terms of sex in the other patients. All 8 patients with nephrotic syndrome diagnosis had one sibling with the same diagnosis and history of parents' consanguinity. Among the 8 patients with nephrotic syndrome, 3 had renal biopsy performed and this was consistent with focal segmental glomerulosclerosis (FSGS). In 5 patients, the CKD diagnosis was steroid-resistance nephrotic syndrome (SRNS). For 12 patients with urolithiasis, 4 had unilateral nephrectomy performed due to staghorn stone. Eight patients had recurrent stone history and all of these patients did not attend regular doctor check-ups until CKD developed. All 7 patients with spina bifida diagnosis were paraplegic and had not attended doctor check-ups after the postnatal defect closed until CKD developed. Five patients with hemolytic uremic syndrome (HUS) diagnosis received the diagnosis in Syria and were monitored in hospital for a period. They were referred to our hospital due to lengthened anuria and the lack of a nephrology clinician in the Syrian hospital and were diagnosed with CKD on arrival. Among these patients, 3 began emergency hemodialysis and 1 began peritoneal dialysis in our hospital. One patient with HUS diagnosis developed CKD in the 2^nd^ year after acute disease. Two patients with HUS diagnosis are still monitored for stage 3 CKD. The distribution of 30 patients with end-stage renal failure in continued dialysis according to diagnosis is given in Table 3. During follow-up, 2 patients receiving hemodialysis were died due to COVID-19 infection.

**Table 1:** Causes of chronic kidney disease

Diagnosis	Number(n)(%)
CAKUT	30 (37.9%)
Urolitiasis	12 (15.1%)
Nephrotic syndrome	8 (10.1%)
Spina bifida (neurogenic bladder)	7 (8.8%)
Hemolytic Uremic Syndrome	6 (7.5%)
Glomerulonephritis	6 (7.5%)
Polycystic kidney	4 (5%)
Cystinosis	2 (2.5%)
Prune-belly syndrome	1 (1.2%)

**Table 2:** Distribution of patients with cakut diagnosis

CAKUT	Number(n) (%)
UP junction obstruction	14 (46.6%)
VUR	9 (30%)
Agenesis	3 (10%)
Multicystic dysplasia	2 (6.8%)
Posterior Uretral Valv	1 (3.3%)

**Table 3:** Distribution and dialysis modalities according to diagnosis of dialysis patients

	Hemodialysis (n)(%)	Peritoneal dialysis (n)(%)
CAKUT	15 (60%)	5 (71.4%)
UP junction obstruction	7 (46.7%)	-
VUR	4 (26.7%)	3 (60%)
Renal agenesis	2 (13.4%)	-
Multicystic dysplasia	1 (6.6%)	1 (20%)
Posterior Uretral Valv	1 (6.6%)	1 (20%)
Hemolytic Uremic Syndrome	3 (12%)	1 (14.3%)
Nephrotic Syndrome	2 (8%)	1 (14.3%)
Cystinosis	1 (4%)	-
Prune belly Syndrome	1 (4%)	-
Unknown etiology	3 (12%)	-
Total	25 (100%)	7 (100%)

When examined in terms of the chosen dialysis modality, the number of patients receiving hemodialysis was significantly higher (25 hemodialysis, 7 peritoneal dialysis). Among the 7 patients with peritoneal dialysis, 5 began peritoneal dialysis in our hospital and 2 were using it on admission. Most patients used hemodialysis due to bad living conditions. Only 2 of the patients in peritoneal dialysis had automatic peritoneal dialysis (APD) applied, with 5 using continuous ambulatory peritoneal dialysis (CAPD). The majority of the 32 dialysis patients did not take medication in addition to dialysis treatment from the time medications given in the clinic ran out until the next check-up due to inability to access medications regularly. Among the 25 patients continuing with hemodialysis, 2 were siblings with ESRD due to vesicoureteral reflux (VUR) and 2 siblings in the family had died with dialysis due to ESRD. The mother was pregnant with her 11^th^ child and had antenatal hydronephrosis diagnosis. The 11^th^ sibling is still followed by our clinic due to posterior urethral valve (PUV) diagnosis. Of the 47 patients with stage 1-4 CKD, 30 did not attend clinic check-ups regularly due to socioeconomic and sociocultural reasons. Thirty-two dialysis patients were recommended for kidney transplant, but the parents of 25 did not like the idea of transplant. Fifteen parents worried about who would look after other children if they were donor, while 10 thought it would be difficult to complete transplant procedures in a foreign country. Four patients who accepted kidney transplant are still waiting for donors. While there were 8 patients on hemodialysis when admitted to our clinic, of the other 24 patients who began dialysis in our hospital, only 7 consented to peritoneal dialysis. The parents of 9 of these 17 patients stated they lived in refugee camps in tents and could not provide the hygiene and social environment for peritoneal dialysis administration and chose hemodialysis, while 8 parents stated they did not want a tube in the abdomen and chose hemodialysis. The comparison of clinical findings and complications in patients with hemodialysis and peritoneal dialysis are given in Table 4. Accordingly, the mean age of patients with peritoneal dialysis was lower. Long-term complications like left ventricle hypertrophy and retinopathy were significantly higher among hemodialysis patients. There was no difference between the groups in terms of hypertension and sex. Among 13 hemodialysis patients with hypertension, 8 used a single, 3 used two, and 2 used three antihypertensives. The 3 peritoneal dialysis patients with hypertension identified were using single antihypertensives. Among the 3 patients with hemodialysis and retinopathy, 2 had stage 2 retinopathy, while 1 had stage 1 retinopathy. During follow-up, 3 of the 7 peritoneal dialysis patients (42.8%) developed at least 1 peritonitis attack, while 18 of the 25 hemodialysis patients (72%) developed catheter infection at least once (*P*<0.05). In both hemodialysis patients with catheter infection and peritonitis patients, gram positive bacteria were most commonly identified, with methicillin-resistant *Staphylococcus aureus* (MRSA) the most frequently isolated bacteria. Catheter infections were the most frequent complications in both groups.

**Table 4:** Comparison of patients with hemodialysis and peritoneal dialysis

	Hemodialysis (n=25)	Peritoneal dialysis (n=7)	*p*
Age (years) (median)(sd)	8.7±3.5(3-16)	5.2±2.7(2-8)	**<0.05**
Age to start dialysis (years)(median)(sd)	3.2±1.8 (1-5)	2.1±1.4 (1-3)	>0.05
Gender n (%)			>0.05
Male	17 ( 68%)	6 (85.7%)	
Female	8 (32%)	1 (14.3%)	
Left ventricule hypertrophy n (%)	16 (64%)	-	**<0.001**
Retinopathy n (%)	3 (12%)	-	**<0.05**
Hypertension n (%)	13 (52%)	3 (42.8%)	>0.05
Dialysis related complications			
Catheter infection	18 (72%)	3 (42.8%)	**p<0.05**
Catheter obstruction	13 (52%)	1 (14.2%)	**p<0.05**
Metabolic complications	2 (8%)	-	**p>0.05**
Hernia	-	1 (14.2%)	**p<0.05**
Dialysis failure	-	-	**p>0.05**

## Discussion

In Turkey, the incidence of CKD in children has increased with the addition of Syrian refugee pediatric patients since 2011.^7^ In our study, obstructive uropathies and congenital kidney diseases were the most common.^9^^,^ 10 When frequency was examined, though similar to the literature, the value was very high (37.9%). This rate varied according to settlement region and sociocultural differences, but was similar to studies in regions where Syrian patients live densely.^10^^,^ 11 While UP stenosis was the most frequently observed obstructive uropathy, the second most common cause of CKD was urolithiasis. Among 12 patients with urolithiasis diagnosis, 8 had history of using baby food made with cows' milk and rice flour apart from breastmilk until 1 year of age. Two patients developed CKD after operation due to staghorn stone. As can be seen, treatable and preventable causes with progression to end-stage renal failure were more frequent. However, the living conditions in this patient group and low socioeconomic levels caused serious setbacks to attending doctors and resulted in diagnostic delays. In this situation, diseases pass the treatable stage and cause permanent structural and functional kidney problems to patients and worse progress to end-stage renal failure leading to dialysis requirements. This situation is extremely upsetting in this century when children's rights are on the agenda.

Among the 79 chronic kidney patients in our study, 32 had end-stage renal failure and continued dialysis treatment. The cause of ESRD was CAKUT for 15 patients (60%) receiving hemodialysis treatment and for 5 patients (71.4%) receiving peritoneal dialysis. Among the 15 CAKUT hemodialysis patients, 8 received diagnosis in the first 3 years of life; however, as they could not attend check-ups, they attended with acute symptoms and began emergency hemodialysis. Of the 5 peritoneal dialysis patients with CAKUT, planned peritoneal dialysis was begun with diagnosis of ESRF during admission to our hospital. However, the majority of these patients could have been saved from CKD with early diagnosis and appropriate treatment. The greatest factor in this delay is low socioeconomic level and poor living conditions. When the choice of dialysis was examined for patients continued dialysis treatment, the number of hemodialysis patients was excessively high (*P*<0.001). Among 7 patients beginning peritoneal dialysis, 5 began in our hospital while only 2 patients were on continued peritoneal dialysis on arrival. Among the 25 patients with hemodialysis, 8 (32%) were undergoing hemodialysis on arrival. Among the 17 patients beginning hemodialysis in our hospital, 3 were referred to our hospital with HUS diagnosis and began emergency hemodialysis. All 14 patients developed ESRF during follow-up and were suitable for peritoneal dialysis. All 14 patients were recommended for peritoneal dialysis, but began hemodialysis as families did not consent. Among parents who rejected peritoneal dialysis, 9 lived in tents in refugee camps and stated they could not provide the hygiene and social environment for peritoneal dialysis application and so chose hemodialysis, while 8 parents stated they did not want a tube in the abdomen and chose hemodialysis.

There is much research investigating the epidemiological and clinical features of Syrian migrant children undergoing hemodialysis treatment due to ESRD. However, the number of studies comparing dialysis outcomes and modalities is very low. The most frequent causes may be that Syrian parents frequently reject peritoneal dialysis due to sociocultural reasons and the inability to access pediatric nephrology experts in the geographical region they live in.^12^ Additionally, studies over many years have shown that for pediatric patients with ESRD, peritoneal dialysis is a more effective method compared to hemodialysis with fewer side effects.^13^^,^ 14 Peritoneal dialysis, including for patients requiring acute dialysis, is a very effective method especially for pediatric patients and is the dialysis of choice with minimal effects on quality of life.^15^^,^ 16 Similarly, peritoneal dialysis is a very reliable treatment choice for pediatric patients in intensive care.^17^ The number of hemodialysis patients in our study was significantly higher compared to those receiving peritoneal dialysis. Studies with Syrian patients have similar rates, while it should not be ignored that nearly all of these studies were performed by pediatric nephrology specialists.^9^^,^ 10 ^,^ 11 It should be considered that the dominant population in our study were migrant patients experiencing difficulties accessing pediatric nephrology experts due to the effects of the war. The most upsetting aspect of this situation is that the patient group most affected by war are children. During follow-up, 3 of the 7 peritoneal dialysis patients developed at least 1 peritonitis attack (42.8%). This rate is similar to other studies.^18^

Four patients continued peritoneal dialysis without problems. There was no patient with kidney transplant, though most hemodialysis patients were recommended for transplant. Only 4 of the 25 hemodialysis patients applied for transplant; however, proceedings did not continue. When the literature is examined, there is no study of the pediatric patient group specifically. As concluded in adult studies, outcomes for Syrian migrant patients with transplant are similar to other patients.^19^^,^ 20 The mean age of patients with hemodialysis and peritoneal dialysis was significantly lower for peritoneal dialysis patients (*P*<0.05). Dialysis durations were similar (*P*>0.05). While clinical complications of left ventricular hypertrophy (*P*<0.001) and retinopathy (*P*<0.05) were significantly higher for hemodialysis patients, hypertension rates were similar (*P*>0.05). This shows that long-term complications of ESRD are observed with higher frequency in hemodialysis. Though complications are related to disease duration, the effect of poor clinical follow-up and sociocultural living conditions in Syrian migrant pediatric patients should not be ignored. The similar follow-up and dialysis durations in peritoneal dialysis and hemodialysis patients in our study group support this view. It is a known fact that peritoneal dialysis is more effective than hemodialysis in the protection of residual renal functions and residual urine amount.^20^ Another reason for the higher prevalence of the mentioned complications in patients undergoing hemodialysis is the rapid decrease in these residual functions. Although there was no significant difference between the two groups in terms of hypertension, hypertension rates in both groups were quite high. This result made us think that adherence to treatment was poor in both groups. In our study, 24 of 32 patients who were followed up on dialysis were started on dialysis in our hospital. Another reason for frequent long-term complications such as left ventricular hypertrophy and retinopathy is that they were not followed up before applying to our hospital and could not receive treatment. However, there is still a need for studies about this topic with higher patient numbers. In both groups the most frequent dialysis complication was catheter infection. There are similar rates in the literature, with MRSA the most frequently isolated vector in both groups (65% hemodialysis, 35% peritoneal dialysis).^21^ This is a more resistant vector compared to other vectors.^22^^,^ 23 Due to recurrent resistant catheter infection, 16 of 25 hemodialysis patients (64%) had history of at least one catheter change. While 3 patients undergoing peritoneal dialysis had peritonitis, they continued peritoneal dialysis after successfully completing treatment.

Though the low number of peritoneal dialysis patients is a limitation of our study, we think that our results will contribute to the literature with good record-keeping and good numbers of Syrian migrant patients with CKD. The lack of acceptance of peritoneal dialysis, a very effective method for ESRD, by families is the most important finding. Due to being a correctable problem, government institutions should perform studies from this aspect, improve living conditions for refugee patients, and bring pediatric patients, the light of the future, back to health and provide healthy individuals for society. Spending sufficient time with these patients to ensure follow-up and suitable treatment choices are performed accurately and regularly will minimize long-term complications of both CKD and ESRD.

Although the examination and treatment possibilities of immigrant patients in our country are not different from our own citizens, socio-educational differences and poor hygiene conditions cause delay in diagnosis and treatment. Being away from their country and psychological-sociological traumas caused by years of war are the biggest reasons for this. Although it is not in our power to change the current war and what is happening in Syria, it should be our main duty to provide equal health and life services to these people who take refuge in our countries for the right to life.

In conclusion, progression to ESRD for preventable reasons is very frequent among CKD patients. The reasons for this include many correctable causes like the population frequently intermarrying, low prenatal diagnosis rate (2.5%), restrictions in access to health organizations, and poor sociocultural and socioeconomic level. Similarly, hemodialysis is preferred for patients with dialysis decision due to ESRD. For more effective use of peritoneal dialysis in this patient group, the government msut improve access opportunities to pediatric nephrologists.

## References

[1] Kaspar CDW, Bholah R, Bunchman TE (2016). A review of pediatric chronic kidney disease. Blood Purif.

[2] Ardissino G, Dacco V, Testa S, Bonaudo R, Claris-Appiani A, Taioli E (2003). Epidemiology of chronic renal failure in children: data from the italkid project. Pediatrics.

[3] Shahdadi H, Sheyback M, Rafiemanesh H, Balouchi A, Bouya S, Mahmoudirad G (2019). Causes of chronic kidney disease in Iranian children: a meta-analysis and systematic review. Ann Glob Health.

[4] North American Pediatric Renal Trials and Collaborative Studies (2011). 2011 Annual dialysis report.

[5] Inker LA, Astor BC, Fox CH, Isakova T, Lash JP, Peralta CA (2014). KDOQI US commentary on the 2012 KDIGO clinical practice guideline for the evaluation and management of CKD. Am J Kidney Dis.

[6] Uwaezuoke SN, Ayuk AC, Muoneke VU, Mbanefo NR (2018). Chronic kidney disease in children: using novel biomarkers as predictors of disease. Saudi J Kidney Dis Transpl.

[7] Akbalik Kara M, Kilic BD, Col N, Ozcelik AA, Buyukcelik M, Balat A (2017). Kidney disease profile of syrian refugee children. Iran J Kidney Dis.

[8] Levey AS, Jong PE, Coresh J, El Nahas M, Astor BC, Matsushita K (2011). The definition, classification, and prognosis of chronic kidney disease: a KDIGO Controversies Conference report. Kidney Int.

[9] Kari JA (2006). Chronic renal failure in children in the Western Area of Saudi Arabia Saudi. J Kidney Dis Transplant.

[10] Saeed MB (2005). The major causes of chronic renal insufficiency in Syrian children: a one-year, single-center experience. Saudi J Kidney Dis Transpl.

[11] Al-Ghwery S, Al-Asmari A (2004). Chronic renal failure among children in Riyadh Military Hospital. Saudi J Kidney Dis Transpl.

[12] Yilmaz M, Aydin N, Dogan C, Turan F, Yilmaz E, Vardar Y (2019). Comparison of the socio-economic situation and living conditions of Syrian and underprivileged Turkish patients receiving hemodialysis. Turk J Nephrol.

[13] Alhusaini O, Wayyani AL, Dafterdar H, Gamlo M, Alkhayat ZA, Alghamdi AS (2019). Comparison of quality of life in children undergoing peritoneal dialysis versus hemodialysis. Saudi Med J.

[14] Woodrow G, Fan S, Reid C, Denning J, Pyrah AN (2017). Renal association clinical practice guideline on peritoneal dialysis in adults and children. BMC Nephrol.

[15] Passadakis PS, Oreopoulos DG (2007). Peritoneal dialysis in patients with acute renal failure. Adv Perit Dial.

[16] Zaritsky J, Warady AB (2011). Peritoneal dialysis in infants and young children. Semin Nephrol.

[17] Bonilla-Félix M (2013). Peritoneal dialysis in the pediatric intensive care unit setting: techniques, quantitations and outcomes. Blood Purif.

[18] Alharthi A (2015). Peritonitis in children with automated peritoneal dialysis. Clin Nephrol.

[19] Sevinc M, Hasbal NB, Ozcelik G, Akinci N, Basturk T, Koc Y (2019). Kidney transplantation outcomes in temporarily protected syrian patients with end-stage renal failure in Turkey. Transplant Proc.

[20] Verrina E, Cappelli V, Perfumo F (2009). Selection of modalities, prescription, and technical issues in children on peritoneal dialysis. Pediatr Nephrol.

[21] Kim JE, Park SJ, Oh JY, Kim JH, Lee JS, Kim PK (2015). Noninfectious complications of peritoneal dialysis in korean children: a 26-year single-center study. Yonsei Med J.

[22] Sahli F, Feidjel R, Laalaoui R (2017). Hemodialysis catheter-related infection: rates, risk factors and pathogens. J Infect Public Health.

[23] Paglialonga F, Esposito S, Edefonti A, Principi N (2004). Catheter related infections in children treated with hemodialysis. Pediatr Nephrol.

